# Medical registrars at the University of the Free State: Burnout, resilience and coping strategies

**DOI:** 10.4102/safp.v65i1.5788

**Published:** 2023-12-15

**Authors:** Lynette J. van der Merwe, Nakedi Motlapema, Tsiu Matsepe, Karabo Nchepe, Pearl Ramachela, Tshilidzi Rangolo, Zizipho Kutu, Gina Joubert, Cornel van Rooyen

**Affiliations:** 1 Division of Health Sciences Education, Faculty of Health Sciences, University of the Free State, Bloemfontein, South Africa; 2 Department of Biostatistics, Faculty of Health Sciences, University of the Free State, Bloemfontein, South Africa

**Keywords:** resilience, burnout, maladaptive coping strategies, adaptive coping strategies, postgraduate training, junior doctor, mental health

## Abstract

**Background:**

Burnout among doctors has been linked with decreased quality of patient care. The coronavirus disease 2019 (COVID-19) pandemic highlighted the need to protect doctors’ mental health and well-being. This study aimed to investigate burnout, resilience and coping strategies among registrars in the MMed programme of the University of the Free State (UFS) in 2020.

**Methods:**

In this quantitative, cross-sectional study, a link to an online anonymous self-administered questionnaire with socio-demographic questions, perceived stress, Copenhagen Burnout Inventory (CBI), Connor-Davidson Resilience Scale and Brief Cope was emailed to all 278 registrars.

**Results:**

Sixty registrars responded (response rate 21.6%). More than half (55.0%) were male and 73.3% were married. There were 28.3% second- and third-year students, respectively. Most (58.3%) had 5–10 years’ work experience. The CBI personal scale had the highest median value (58.3; interquartile range [IQR]: 43.3; 70.8) with 70% scoring ≥ 50. The median score for resilience was 78 of 100 (IQR: 69; 84). There were weak negative correlations between resilience and burnout scores (*r* = –0.31 to *r* = –0.37). Planning, positive reframing and acceptance were the most frequently used adaptive coping mechanisms; self-distraction was the most frequently used maladaptive coping mechanism. There was no association between gender and burnout and resilience scores.

**Conclusion:**

Registrars were resilient with low levels of patient- and work-related burnout, and higher personal burnout, using mostly positive coping strategies.

**Contribution:**

This study gives insight into the well-being of registrars at the UFS during COVID-19. Continuous monitoring and support for this population are essential to foster mental health and well-being.

## Introduction

Resilience is one of the qualities essential to doctors in their profession. Cooke et al.^1^ defined resilience as ‘a dynamic, evolving process of positive attitudes and practical strategies to respond to life stressors’. Doctors’ resilience is tested continually because of daily stressors. Ineffective management of workplace stress increases the risk of experiencing burnout in different aspects of life, including the workplace, home and relationships.^2,3^ During the coronavirus disease 2019 (COVID-19) pandemic, the importance of resilience to protect against distress was highlighted in a 2020 Belgian study among healthcare workers.^4^

Studies on the effects of burnout on doctors’ personal lives showed an association with alcohol abuse, relationship stress, disease and motor vehicle accidents.^5,6^ Qualities necessary for personal development, such as concentration, honesty, integrity, empathy, altruism, self-regulation and professional behaviour, may also be affected.^5,6^

According to Rossouw et al.,^7^ burnout is:

[*A*] persistent, negative, work-related state of mind in ‘normal’ individuals, that is primarily characterised by exhaustion, and is accompanied by distress, a sense of reduced effectiveness, decreased motivation and the development of dysfunctional attitudes and behaviour at work. (p. 567)

According to the World Health Organization (WHO) 11th revision of the International Classification of Diseases (ICD), burnout is an occupational phenomenon, not a medical condition.^3^ The characteristics of burnout are emotional exhaustion (feeling depleted, weary or without energy), depersonalisation (feelings of negativism or cynicism at work or irritability towards patients) and a low level of personal accomplishment resulting in reduced professional efficacy, low productivity and dissatisfaction.^2,3^

The global COVID-19 pandemic increased the risk of doctors experiencing burnout and emphasised the need to protect and optimise their mental health and well-being. An analysis of 12 studies in China and one in Singapore during the COVID-19 outbreak showed that the prevalence of anxiety, depression and insomnia among healthcare professionals was 23.2% and 38.9%, respectively.^8^ These findings highlight that the impact of the pandemic on doctors’ psychological health is related to escalated levels of burnout. A survey from 2012 to 2014 of 1701 registrars in the United States (US) found a burnout level of 60.3%.^5^ Burnout is associated with depression and anxiety, although these are not overlapping constructs.^6,9^ Harvey et al.^6^ highlighted that the mental health and well-being of physicians should be better protected, especially in the post-pandemic context.

Several studies have been conducted on burnout among healthcare professionals in South Africa. At the University of the Witwatersrand School of Clinical Medicine, burnout was found among 84% of the registrars (data collection date unknown, publication 2019).^10^ Another study among anaesthesiologists working at the University of the Witwatersrand showed high levels of burnout in 21.0% of the participants (data collection 2013, publication 2015).^11^ In Cape Town community healthcare clinics and district hospitals in the Western Cape, 76% of the doctors were reported to experience burnout, and 81% of doctors in rural hospitals were reported to experience burnout (data collection 2010, publication 2013).^7^ At the University of the Free State (UFS) (data collection 2012–2013, publication 2013), Sirsawy et al.^12^ reported that 15.6% of registrars and medical officers working in Bloemfontein public healthcare facilities had a high degree of burnout on all three subscales of the Maslach Burnout Inventory (MBI). As cautioned in a systematic review by Rotenstein et al.,^13^ determining the prevalence of burnout as a consequence of work-related stress remains elusive due to the absence of standardised measurement tools, poor consensus regarding definition and erratic study quality.

Developing risk-taking behaviours and negative coping strategies are evident in healthcare professionals. Concern about the prevalence of substance misuse among physicians has been reported. In the United States, a study among 7209 American physicians reported that 15.3% had scores indicating alcohol abuse or alcohol dependence.^14^ Similarly, in the United Kingdom (UK) and Denmark, the prevalence of risky substance use between 5% and 20% was reported.^15,16^ An Australian survey found no significant differences between physicians in various specialities regarding alcohol use.^17^ Evidence among South African specialists is scarce. However, among South African anaesthesiologists, lifetime use of alcohol (92.8%), tobacco (42.3%), cannabis (34.7%) and sedatives (34.4%) has been reported. These healthcare practitioners may be at moderate risk for substance abuse, implying possible damaging or destructive use.^18^

Focusing on planning and solving the problems faced rather than emotional responses seem to promote care without depersonalisation in situations of extreme stress.^19,20^ Strategies and interventions to improve mental health among physicians at the health system level are essential, especially in the early career stages.^6^

A registrar, sometimes referred to as a specialist registrar, is a junior doctor who has completed their foundation training but is still training in a speciality area of medicine.^21^ Specialist training in Master of Medicine (MMed) programmes at South African universities exposes doctors to the rigours of postgraduate study and clinical service delivery, often in stressful environments. Suboptimal conditions may lead to poor training quality of future specialists. Therefore, it is essential to obtain information regarding burnout, resilience and coping strategies among healthcare practitioners. Registrars were among those at high risk during the COVID-19 pandemic response because of increased workload and risk of exposure, increasing their vulnerability to mental ill health.

This study aimed to investigate burnout, resilience and coping strategies among registrars in the MMed programme at the UFS. The impact of gender on burnout and resilience was assessed.

## Methods

### Study design

This was a quantitative cross-sectional study.

### Target population and sample

The target population was all registrars registered in the MMed programme at the Faculty of Health Sciences, UFS, in 2020. There are 27 departments in the Schools of Clinical Medicine and Pathology, respectively, that train registrars. In the second semester of 2020, there were 278 registrars in the programme (Ms M. du Randt, e-mail communication with Postgraduate Administration Office, Faculty of Health Sciences). There were no exclusion criteria.

### Measurements

Data were collected by a self-administered, voluntary, anonymous online questionnaire available in English. The Evasys online survey system was used as the platform for the electronic questionnaire. A link to the questionnaire was sent via the Postgraduate Office to all registrars from 14 August 2020 until 30 November 2020, with reminders every 2 weeks to encourage participation. The questionnaire included a demographic information section and questions regarding current perceived stress and three validated instruments to determine burnout, resilience and coping strategies.

### Demographic and background information

Demographic data included age, gender and marital status, field of speciality, year of study in the MMed programme, year when undergraduate qualification was obtained, and questions determining current stress levels, major life events and stressors were posed.

### Burnout

The *Copenhagen Burnout Inventory* (CBI)^22^ comprises of 19 items that measure three areas of burnout: personal burnout (6 items), work-related burnout (7 items) and patient-related burnout (6 items). Items are rated on a 5-point Likert scale regarding the experience of burnout: ‘always or to a very high degree’ (score 100), ‘often or to a high degree’ (score 75), ‘sometimes or somewhat’ (score 50), ‘seldom or to a lower degree’ (score 25) and ‘never or to a very low degree’ (score 0). One item has reverse scoring. A mean score out of 100 is calculated, per scale, with higher scores indicating burnout. The CBI is a validated, free, publicly available (open access) and reliable instrument to determine burnout.^23^

### Resilience

The *Connor-Davidson Resilience Scale* (CD-RISC)^24^ measures resilience. It comprises 25 items rated on a 5-point Likert scale with responses from ‘not true at all’ (score 0) to ‘true nearly all of the time’ (score 4). The total score ranges from 0 to 100, with higher scores indicating higher resilience. The CD-RISC has been found to be a reliable, valid measurement tool in the South African context.^25^

### Coping mechanism

The *Brief Coping Orientation to Problems Experienced Inventory* (COPE)^26^ measures 14 coping styles with 2 questions per coping style rated on a 4-point Likert scale with responses from ‘I haven’t been doing this at all’ (score 1) to ‘I have been doing this a lot’ (score 4). Per coping style scores range from 2 (not done this) to 8 (doing this a lot). Coping styles are grouped as adaptive (active coping, planning, positive reframing, acceptance, emotional support, instrumental support, humour, religion) or maladaptive (self-distraction, self-blame, denial, behavioural disengagement, substance use, venting). The use of the Brief COPE has been validated and indicated as suitable across cultures.^27^

### Pilot study

A pilot study was conducted in August 2020 on five respondents from other postgraduate programmes in the Faculty of Health Sciences, UFS, in 2020 to test the electronic survey link and administration process, time needed for completion and clarity of instructions. No changes were required to the questionnaire. Data from the pilot study were excluded from the main study.

### Statistical analysis

The questionnaire required no coding by the research team as data were available in an Excel spreadsheet through the Evasys system. Data were analysed by the Department of Biostatistics, Faculty of Health Sciences, UFS, using SAS Version 9.4. Descriptive statistics included frequencies and percentages (categorical variables) and medians and interquartile ranges (IQRs) (numerical variables). Associations between categorical variables were investigated using chi-square and Fisher’s exact tests. Numerical variables in subgroups were compared using Mann-Whitney and Kruskal-Wallis tests. Spearman’s rank correlations assessed the correlation between numerical variables. *P*-values < 0.05 were considered statistically significant.

### Ethical considerations

The protocol was approved by the Health Sciences Research Ethics Committee, Faculty of Health Sciences, UFS (UFS-HSD2017/0570/3006) and the Free State Department of Health. The questionnaire was anonymous, and no personal identifiers were captured to ensure confidentiality. Participation was voluntary, and consent was implied through participation. No reward or incentive for participation was included.

## Results

Sixty respondents completed the online survey (response rate 21.6%). Their median age was 34 years (range: 27–52 years). As summarised in Table 1, 55.0% of the respondents were male, and 73.3% were married. The highest percentage of respondents were either second- (28.3%) or third-year registrars (28.3%). According to the year that the respondents obtained their medical degree, 35 (58.3%) had 5–10 years of work experience and 25 (41.7%) had > 10 years of work experience.

**TABLE 1:** Demographic details of respondents (*N* = 60).

Variable	*n*	%
**Gender**		
Male	33	55.0
Female	27	45.0
**Marital status**		
Single	11	18.3
Married	44	73.3
Divorced	3	5.0
Other	2	3.3
**Year of study in MMed programme**		
First	12	20.0
Second	17	28.3
Third	17	28.3
Fourth	13	21.7
Fifth	1	1.7

MMed, Master of Medicine.

Of the 27 departments, respondents from 18 departments completed the online questionnaire. Respondents were mainly from the School of Clinical Medicine (*n* = 52, 88.1%), with the Department of Anaesthesia having the highest number of respondents (*n* = 14). Seven respondents (11.9%) were from the School of Pathology, and one did not indicate a department.

### Stressors

Major life events in the previous 12 months reported by the respondents included major illness in a family member (*n* = 19, 31.7%), personal health event or major illness (*n* = 17, 28.3%), death of a close friend or family member (*n* = 15, 25.0%), relationship breakup (*n* = 5, 8.3%) or divorce (*n* = 1, 1.7%).

When rating their current level of stress, 33 (55.0%) respondents reported mild stress, 19 (31.7%) severe to debilitating stress, 5 (8.3%) had minor levels of stress and 3 (5.0%) reported no current stress.

Respondents were asked to indicate any current major stressors. The majority (*n* = 51, 85.0%) noted academic stressors, followed by personal (*n* = 27, 45.0%) and financial (*n* = 19, 31.7%) stressors. Only four respondents noted no current stressors. Six (10.0%) respondents reported ‘other’ stressors related to COVID-19, criminal cases, mental health issues and lack of support at home.

Respondents rated the current level of satisfaction with different support structures. Respondents were satisfied to very satisfied with family (*n* = 33, 71.7%) and friends and peers (*n* = 36, 60.0%) but neutral towards the Faculty of Health Sciences (*n* = 25, 41.7%) and UFS (*n* = 34, 56.7%). Regarding the learning environment at the faculty, the highest percentage of respondents were neutral on both the statement that holistic support is available (*n* = 23, 38.3%) and that they are satisfied with the learning environment (*n* = 22, 36.7%).

### Resilience

Most respondents (*n* = 54, 90.0%) reported high self-perceived levels of resilience. This was supported by the median CD-RISC score of 78 out of 100 (IQR: 69; 84). Respondents with high self-perceived levels of resilience had higher scores on the CD-RISC (median: 78, IQR: 70; 84) than respondents with low self-perceived levels of resilience (median: 67, IQR: 43–83) (*p* = 0.13).

### Burnout

As shown in Table 2, the highest median score was on the personal scale (58.3), with 70.0% of respondents scoring ≥ 50. The lowest median was on the patient scale (29.2), with 20.4% of respondents scoring ≥ 50.

**TABLE 2:** Copenhagen burnout inventory burnout scores.

Scale	Respondents *(n)*	Median	IQR	Score ≥ 50	
				*n*	%
Personal burnout	60	58.3	43.3; 70.8	42	70.0
Work-related burnout	60	53.6	39.3; 76.8	35	58.3
Patient-related burnout	54	29.2	12.2; 45.8	11	20.4

IQR, interquartile range.

### Coping mechanisms

Adaptive coping mechanisms generally had higher median scores (i.e., were used more frequently) than maladaptive coping mechanisms (Table 3). Planning, positive reframing and acceptance were the most frequently used adaptive coping mechanisms (median: 6), and self-distraction was the most frequently used maladaptive coping mechanism (median: 5). Behavioural disengagement, denial and substance use were the least used maladaptive coping mechanisms.

**TABLE 3:** Median scores for different coping mechanisms in Brief Cope.

Coping mechanism	Median†	IQR
**Adaptive coping**		
Active coping	5	4; 6
Planning	6	4; 6
Positive reframing	6	4; 6
Acceptance	6	5; 7
Emotional support	5	4; 6
Instrumental support	4	4; 6
Humour	5	3; 6
Religion	5	4; 7
**Maladaptive coping**		
Self-distraction	5	4; 6
Self-blame	4	3; 5
Denial	2	2; 3
Behavioural disengagement	2	2; 4
Substance use	2	2; 3.5
Venting	4	3; 5

IQR, interquartile range.

† , On a scale from 2 (‘do not do this at all’) to 8 (‘do this a lot’).

### Associations

There were weak negative correlations between the resilience score and burnout scores (Table 4). Personal and work-related burnout scales were strongly correlated (*r* = 0.87, *p* < 0.01), but the correlations between these two scales and the patient-related scale were weak (*r* < 0.30).

**TABLE 4:** Correlations between resilience and burnout scores.

Scale	Resilience (CD-RISC)	CBI personal	CBI work-related	CBI patient-related
**Resilience (CD-RISC)**				
*r*	1.00	−0.37	−0.35	−0.31
*p*	-	0.01	0.01	0.03
*n* respondents	55	55	55	50
**CBI personal**				
*r*	-	1.00	0.87	0.28
*p*	-	-	< 0.01	0.04
*n* respondents	-	60	60	54
**CBI work-related**				
*r*	-	-	1.00	0.23
*p*	-	-	-	0.10
*n* respondents	-	-	60	54

CBI, Copenhagen Burnout Inventory; CD-RISC, Connor-Davidson Resilience Scale; *r*, correlation coefficient.

There were no statistically significant differences between genders regarding resilience and burnout scores (Table 5).

**TABLE 5:** Burnout and resilience scores in males and females.

Scale	Male			Female			*p*
	*n*	Median	IQR	*n*	Median	IQR	
Resilience (CD-RISC)	33	78	68.0; 83.0	27	76.5	70.0; 84.0	0.93
CBI personal	33	58.3	45.0; 75.0	27	62.5	41.7; 70.8	0.99
CBI work-related	33	50.0	39.3; 71.4	27	53.6	39.3; 78.6	0.96
CBI patient-related	32	29.2	4.2; 45.8	22	29.2	12.5; 45.8	0.55

CBI, Copenhagen Burnout Inventory; CD-RISC, Connor-Davidson Resilience Scale; IQR, interquartile range.

## Discussion

This study aimed to investigate the level of burnout, resilience and coping strategies among registrars in the MMed programme at UFS. There was a response rate of 21.6%. A study done at the University of the Witwatersrand had a response rate of 34%,^10^ while response rates of 66.8% – 68.3% were reported in studies using the MBI in South African doctors.^11,12^ This study’s low response rate could be attributed to the COVID-19 pandemic in the country at the time of data collection. Data were collected between August 2020 and November 2020, when the country was on Level 2 and Level 1 alert^28^ with varying restrictions in place and therefore differences in patient profiles and disease presentations at public healthcare facilities. The population of registrars taking part in this research were the first line of defence in the healthcare response to the pandemic, which would have impacted their availability to participate. Reminders were sent every 2 weeks encouraging participation, and the respective heads of departments were contacted individually to enlist their support. No incentives were provided.

According to the CD-RISC scores, respondents’ resilience was generally high. In the study population, the median score was 78, which is higher than the average resilience score of 65 reported by a study in the UK.^29^ Although there is no population norm score, high scores indicate a high level of resilience. The manual of the CD-RISC includes tables of mean CD-RISC scores from other published studies.^29^ These findings provide valuable information about resilience among doctors working in LMICs (lower- and middle-income countries) who are potentially under greater strain because of additional burdens of resource constraints and workforce shortages. Although this population of early career specialists in training may seem resilient, the responsibility of the healthcare system and its professional regulatory bodies in supporting doctors’ mental health and well-being should not be ignored.^6^

The total percentage of burnout was lower than the 84% reported in a study published in 2019 at the University of the Witwatersrand using the MBI definition^10^ but higher than the 26.3% high burnout found in a previous study published in 2013 among registrars and medical officers in Bloemfontein.^12^ The findings regarding burnout in this study could have been affected by the sample size of 60 and the fact that some departments were not well represented, as well as by the simultaneous COVID-19 pandemic. The study findings could have been influenced through non-participation by those experiencing burnout or low resilience. The relationship between burnout and individual characteristics such as gender, age and marital status remains inconclusive.^30,31,32^ In this study, there was no significant difference between male and female respondents regarding resilience and burnout scores, similar to a study in the UK.^29^

An individual’s approach to life stressors is essential to determine what impact the stressor will have on that person. Stress and depression could be predicted by the choice of coping mechanisms when experiencing a stressor.^33^ Registrars face a heavy workload, as well as academic and personal stress, increasing their vulnerability to mental ill health.^6^ Changes in individual burnout levels may impact organisational culture and patient care, leading to poor physical and mental health among doctors.^6^

In this study, respondents reported using adaptive strategies more frequently than maladaptive strategies, notably planning, positive reframing and acceptance. In contrast, in a UK study, self-distraction, a maladaptive coping strategy, was the most frequently reported.^29^ Behavioural disengagement, substance abuse and denial were the least frequently reported coping mechanisms in this study population. These results correspond with two other studies where substance use and denial had the lowest mean values compared to other coping strategies.^29,34^ However, continuous changing demands on healthcare workers and the lasting sequelae of dealing with crises, for example, the COVID-19 pandemic, may place individuals at higher risk in the future for resorting to maladaptive coping strategies.^6^

The findings reporting low use of substances as a coping strategy could be because of fear of victimisation by employers when self-disclosing the use of potentially harmful substances. It is crucial to encourage the practice of healthy coping strategies to develop resilience against life stressors. However, the responsibility for a physician’s mental well-being cannot be expected from the individual alone but should be shared at system and organisational levels.^6^ Coping with changes in the healthcare environment characterised by heavy workload and limited resources should be addressed by various stakeholders at all levels of the healthcare system and regulatory bodies. Such interventions should be integrated and evidence-based, focusing on individual needs and systemic issues.^6^

### Limitations

The response rate and the concurrent COVID-19 pandemic were major limitations in this study. Email accounts could have been inactive, or the respondents may not have opened emails in time to participate in the study. Because of *Protection of Personal Information Act* (POPI Act)^35^ restrictions, alternative messaging platforms that may have increased the response rate, for example, WhatsApp, had not been used. The small sample size because of the low response rate affected the results, and some subgroup analyses, such as differences between specialities, could not be performed.

## Conclusion and recommendations

Despite the small population, this cross-sectional study provides valuable insight into mental health among early career doctors participating in specialist training in an LMIC during the COVID-19 pandemic. Registrars in the MMed programme at the UFS had high resilience and low patient- and work-related burnout and mainly used adaptive coping strategies to deal with stressors, despite higher personal burnout scores. A bigger sample size with a well-diversified and well-represented population is recommended to yield more conclusive results. This study contributes to the growing body of knowledge regarding burnout, resilience and coping among the healthcare workforce. Increased awareness of the need to prioritise doctors’ mental health and well-being in a post-COVID-19 context and in the face of persistent challenges is imperative.
